# Germline *BRCA1* ‐Mutated Synchronous and Metachronous Pancreatic Acinar Cell Carcinoma With Long‐Term Survival

**DOI:** 10.1002/cnr2.70007

**Published:** 2024-08-26

**Authors:** Tomohiro Kubo, Yuki Ikeda, Joji Muramatu, Kazuma Ishikawa, Makoto Yoshida, Kazuharu Kukita, Masafumi Imamura, Shintaro Sugita, Akihiro Sakurai, Kohichi Takada

**Affiliations:** ^1^ Department of Medical Oncology Sapporo Medical University School of Medicine Sapporo Hokkaido Japan; ^2^ Department of Surgery, Surgical Oncology and Science Sapporo Medical University School of Medicine Sapporo Hokkaido Japan; ^3^ Department of Surgical Pathology Sapporo Medical University School of Medicine Sapporo Hokkaido Japan; ^4^ Department of Medical Genetics and Genomics Sapporo Medical University School of Medicine Sapporo Hokkaido Japan

**Keywords:** germline *BRCA1* mutation, HBOC, pancreatic acinar cell carcinoma, synchronous and metachronous tumor

## Abstract

**Background:**

Pancreatic acinar cell carcinoma (PACC) is a rare pancreatic neoplasm. Recently, molecular analysis revealed that PACC shows a high frequency of the *BRCA*1/2 mutation and is likely to be considered a cancer associated with hereditary breast and ovarian cancer (HBOC). Hereditary cancers, including HBOC, are characterized by multifocal and/or metachronous tumors. However, no case reports exist of germline *BRCA1*‐mutated synchronous and metachronous PACC.

**Case:**

A 58‐year‐old man was diagnosed with synchronous and metachronous PACC at the age of 56 and underwent two surgeries. Ten months after the second surgery, the patient developed multiple liver metastases. Gemcitabine plus nab‐paclitaxel therapy was administered as first‐line chemotherapy. After seven cycles, computed tomography examination revealed progressive disease (PD). Therefore, modified FOLFIRINOX (mFFX) was administered as second‐ line chemotherapy. After 19 cycles of mFFX, comprehensive cancer genomic profiling (CGP) identified a *BRCA1* pathogenic variant that was confirmed to be germline origin. Accordingly, we treated the patient with olaparib; however, he was diagnosed with PD after 4 months. He subsequently died 5 years and 9 months after the initial surgery, and 3 years and 10 months after chemotherapy. Based on the genetic data of the patients, his family members received genetic counseling followed by cascade testing. Consequently, the same g*BRCA1* pathogenic variant was detected in the son and his surveillance for HBOC‐related cancers was initiated.

**Conclusion:**

We diagnosed a 58‐year‐old man with a synchronous and metachronous PACC with germline *BRCA1* pathogenic variant. Considering that PACC is likely to have *BRCA1/2* mutations responsible for HBOC, we need to be aware of the possible presence of multifocal and/or metachronous tumors in patients with PACC. Additionally, patients with PACC should undergo genetic examinations, which would be beneficial in determining treatment strategies and health care for blood relatives.

## 1 Introduction

Pancreatic acinar cell carcinoma (PACC) accounts for approximately 1% of pancreatic neoplasms and is considered a relatively rare tumor [1]. Several differences exist, in terms of biological behaviors and gene signatures, between PACC and pancreatic ductal adenocarcinoma (PDAC). Recent reports have shown that PACC has a distinctive mutational landscape [2, 3, 4, 5]. PACC exhibits a higher frequency of *BRCA1/2* mutations compared to PDAC and is more likely to be an associated cancer of hereditary breast and ovarian cancer syndrome (HBOC). Hereditary cancers, including HBOC, are characterized by multifocal and/or metachronous tumors. Although PACC should be considered as part of the spectrum of *BRCA1/2*‐related malignancies, reports do not exist of *BRCA*‐mutated synchronous and metachronous PACC. We previously reported on this case as being the first instance of PACC that was found to be both synchronous and metachronous [6]. Subsequent genetic testing revealed the presence of a germline *BRCA1* mutation, proving that this was an HBOC‐associated PACC. Herein, we highlight this PACC case as it suggests that aggressive genetic testing is required for this type of pancreatic cancer.

## 2 Case

**FIGURE 1:**
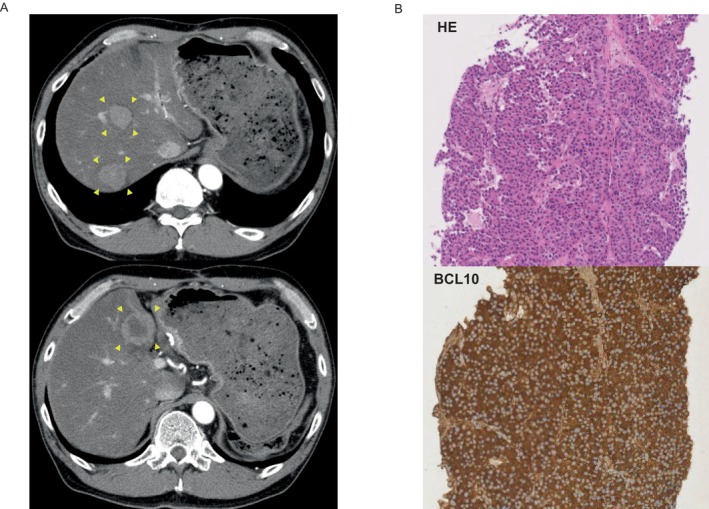
Contrast‐enhanced abdominal computed tomography and pathological findings of liver metastases. (A) Contrast‐enhanced abdominal computed tomography (CT) revealed three hypervascular masses (yellow arrowheads). (B) A percutaneous liver biopsy specimen was positive for BCL10, revealing the presence of PACC. BCL10, B‐cell lymphoma/leukemia 10; CT, computed tomography; HE, Hematoxylin eosin; PACC, pancreatic acinar cell carcinoma.

**FIGURE 2:**
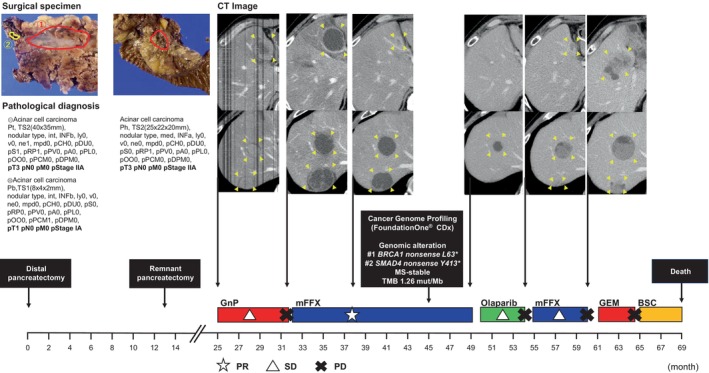
Clinical course after initial diagnosis. BSC, best supportive care; CT, computed tomography; FFX, fluorouracil, irinotecan, and oxaliplatin; mFFX, modified FFX; GEM, gemcitabine; GnP, gemcitabine and nab‐paclitaxel; MS, microsatellite status; PD, progressive disease; PR, partial response; SD, stable disease; TMB, tumor mutational burden.

**FIGURE 3:**
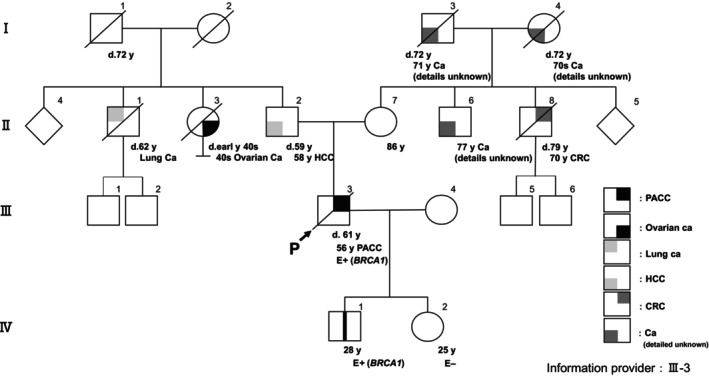
The patient's family trees showing cases with germline *BRCA1* variants. Ca, cancer; CRC, colorectal cancer; d, death; E, evaluation; HCC, hepatocellular carcinoma; P, proband; PACC, pancreatic acinar cell carcinoma.

A 58‐year‐old man was previously diagnosed with PACC of the pancreatic tail at the age of 56 and underwent a distal pancreatectomy; two masses were identified in the caudal and body of the pancreas at Sapporo Medical University Hospital (Sapporo, Japan) in March 2017. Ten months after surgery, a residual recurrence was found in the pancreatic head. The patient subsequently underwent a total resection of the residual pancreas at the same hospital in April 2018. We have previously reported details of the clinical course of the cancer [6]. Ten months after the second surgery, an abdominal computed tomography (CT) scan revealed three hypervascular masses in the liver. The hepatic tumor was diagnosed as a metastatic PACC based on the pathological analysis of a targeted hepatic tumor biopsy (Figure 1). Gemcitabine plus nab‐paclitaxel (GnP) therapy (gemcitabine, 1000 mg/m^2^ days 1, 8, 15; nab‐paclitaxel, 125 mg/m^2^ days 1, 8, 15; every 4 weeks) was administered as first‐line chemotherapy. After seven cycles, CT examination revealed the tumor had changed into a hypovascular tumor but its size had increased. We judged this to be PD. A modified combination regimen of 5‐fluorouracil, leucovorin, oxaliplatin, and irinotecan (modified FOLFIRINOX [mFFX]) therapy [5‐fluorouracil, 2400 mg/m^2^ days 1 and 2; leucovorin, 200 mg/m^2^ day 1; oxaliplatin, 85 mg/m^2^ day 1; irinotecan, 150 mg/m^2^ day 1; every 2 weeks] was administered as second‐line chemotherapy. The best response was a partial response, resulting in a 78% reduction. After 19 cycles of mFFX, comprehensive cancer genomic profiling (CGP; FoundationOne CDx) was performed on the second set of surgical specimens in order to explore effective therapeutic options according to gene alterations. Pathogenic variants were found in *BRCA1* (c.188T>A, L63*, allele frequencies: 60.64%) and *SMAD4* (c.1239C>A, Y413*, allele frequencies: 16.01%). The patient showed a family history of HBOC‐related cancers: For example, his paternal aunt died in her 40s due to ovarian cancer. The same *BRCA1* pathogenic variant was detected in the germline of the patient by a single‐site sequencing; as a result, he was diagnosed with HBOC. After Japanese public health insurance covered treatment with olaparib, the patient was switched to this at a dose of 600 mg/day. After 4 months of treatment, the patient was judged to have PD. He was then treated with a reduced dose of mFFX (5‐fluorouracil, 1800 mg/m^2^ days 1 and 2; leucovorin, 200 mg/m^2^ day 1; oxaliplatin, 65 mg/m^2^ day 1; irinotecan, 120 mg/m^2^ day 1; every 2 weeks), but the patient was judged to have PD after 5 months. Subsequently, he was treated with gemcitabine monotherapy (gemcitabine, 1000 mg/m^2^ days 1, 8, 15 every 4 weeks); a response was not observed. The patient died 5 years and 9 months after his initial surgery, and 3 years and 8 months after the initiation of chemotherapy (Figure 2). His family members received genetic counseling and subsequent cascade genetic testing. The son and daughter underwent single‐site sequencing and the same g*BRCA1* pathogenic variant was detected in the patient's son, prompting the initiation of cancer surveillance thereafter (Figure 3).

## 3 Discussion

PACC has genetic features that are distinct from PDAC. In one study, up to 45% of PACC cases had a deficiency of DNA damage repair pathway genes such as *BRCA1/2* [2]. Pathogenic variants in g*BRCA1/2* are implicated in HBOC, which predisposes individuals to cancers such as those of the breast, ovary, pancreas, and prostate. The cumulative risk for developing pancreatic cancer by 70 years of the age is between 1% and 4% for carriers of g*BRCA1/2* pathogenic variants [7]. Pathogenic variants in DNA damage repair gene mutations, such as *ATM*, *PALB2*, and *CHEK2*, have also been shown to be associated with HBOC [8, 9, 10]. Thus, PACC should be considered as part of the spectrum of HBOC‐related malignancies with the potential for being both synchronous and metachronous cancers. However, no prior reports exist of synchronous and metachronous *BRCA1/2*‐mutated PACC. To our knowledge, this case is the first such report.

The prognosis of postoperative recurrence or unresectable PACC remains poor, similar to that of PDAC [11]. Accumulated evidence has shown that *BRCA‐*mutated tumor cells are sensitive to platinum analog chemotherapeutics and poly ADP ribose polymerase (PARP) inhibitors [12]. Specifically, the efficacies of platinum‐based chemotherapy and PARP inhibitors for *BRCA*‐mutated pancreatic cancer, including PACC, have already been reported [13, 14]. In fact, the total duration of response in our case was approximately 14 months with platinum‐based chemotherapy and PARP inhibitors, resulting in long‐term survival. In addition to *BRCA1/2* mutations, PACC has also been reported to have other druggable gene mutations, such as *PALB2*, *ATM*, *BRAF*, and *JAK1* [15]. Currently, few reports exist on the efficacy of genotype‐matched therapy for *PALB2*, *ATM*, *BRAF*, and *JAK1* mutations, with the exception of BRCA in PACC, with only case reports having achieved a CR with BRAF and MEK inhibitors for *BRAF* V600E [16]. Although the efficacy of genotype‐matched therapy for PACC has to first be established in clinical trials, such treatments may be good candidates for a personalized approach to therapy based on the gene mutations in PACC.

Genetic counseling to assess a patient's genetic predisposition and to plan for future healthcare may be not beneficial for those with unresectable pancreatic cancer and a poor‐prognosis. However, surveillance, risk‐reducing surgery, and oncologic treatment for gBRCA1/2 carriers have been reported to be useful in health care [17, 18, 19]. Like this case, assessing the genetic predisposition of a patient's family members based on the results of the patient's g*BRCA1/2* pathogenic variant is useful in guiding the surveillance of potential carriers.

In conclusion, we diagnosed a 58‐year‐old man with a synchronous and metachronous PACC that was subsequently found to have a g*BRCA1* pathogenic variant. Considering PACC is likely to be positive for *BRCA1/2* mutations responsible for HBOC, resectable cases should be closely followed up with the prospect that the PACC may be synchronous or metachronous cancer. In both resectable and unresectable cases, genetic tests should be performed to assist in healthcare, including cancer surveillance, for blood relatives. Furthermore, in unresectable cases, genetic tests may lead to assisting in the selection of chemotherapeutic regimens and may contribute to a prolonged prognosis.

## Author Contributions

**Tomohiro Kubo:** conceptualization, investigation, writing – original draft, resources, visualization. **Yuki Ikeda:** investigation, resources. **Joji Muramatu:** resources. **Kazuma Ishikawa:** resources. **Makoto Yoshida:** resources. **Kazuharu Kukita:** resources. **Masafumi Imamura:** resources. **Shintaro Sugita:** resources. **Akihiro Sakurai:** writing – review and editing. **Kohichi Takada:** writing – review and editing, conceptualization, supervision.

## Ethics Statement

The authors have obtained informed consent from the individual involved prior to this study.

## Conflicts of Interest

The authors declare no conflicts of interest.

## Data Availability

Due to the nature of this research, participants of this study did not agree for their data to be shared publicly, so supporting data is not available.
